# A Differential Pressure Instrument with Wireless Telemetry for *In-Situ* Measurement of Fluid Flow across Sediment-Water Boundaries

**DOI:** 10.3390/s90100404

**Published:** 2009-01-09

**Authors:** Alan T. Gardner, Hanan N. Karam, Ann E. Mulligan, Charles F. Harvey, Terence R. Hammar, Harold F. Hemond

**Affiliations:** 1 Department of Applied Ocean Physics and Engineering, Woods Hole Oceanographic Institution, 266 Woods Hole Rd, Woods Hole, MA, 02543, USA; 2 Department of Civil and Environmental Engineering, Bldg 48, Massachusetts Institute of Technology, Cambridge, MA 02139; 3 Marine Policy Center, Woods Hole Oceanographic Institution, 266 Woods Hole Rd, Woods Hole, MA, 02543, USA

**Keywords:** Pressure sensor, wireless, hydrology, data logger, oceanographic instrumentation

## Abstract

An instrument has been built to carry out continuous *in-situ* measurement of small differences in water pressure, conductivity and temperature, in natural surface water and groundwater systems. A low-cost data telemetry system provides data on shore in real time if desired. The immediate purpose of measurements by this device is to continuously infer fluxes of water across the sediment-water interface in a complex estuarine system; however, direct application to assessment of sediment-water fluxes in rivers, lakes, and other systems is also possible. Key objectives of the design include both low cost, and accuracy of the order of ±0.5 mm H_2_O in measured head difference between the instrument's two pressure ports. These objectives have been met, although a revision to the design of one component was found to be necessary. Deployments of up to nine months, and wireless range in excess of 300 m have been demonstrated.

## 1. Introduction and Background

### 1.1. The measurement of fluid flow across the sediment-water boundary

Flow of water between surface water bodies and groundwater is a topic of much scientific and practical interest. In marine environments these flows are sometimes referred to by the terms ‘submarine groundwater discharge’ (SGD) and ‘submarine groundwater recharge’ (SGR). Examples of water flow across the sediment-aquifer boundary include saltwater intrusion into coastal aquifers, exchange of water between estuaries and sediments, groundwater discharge to rivers or streams, aquifer recharge from rivers induced by aquifer pumping, and groundwater inflow or outflow to lakes. The phenomenon is important both because it transports dissolved chemicals and because of the unique chemical and biological conditions that are created at the boundary between natural water bodies and their associated soils and sediments.

Many techniques are employed to measure or estimate SGD and SGR. Direct measurements can be made using seep meters, which can be done either manually (e.g., [1]) or automatically (e.g., [2]). The flow of water can also be estimated by observing the change in concentration of tracers, such as naturally occurring trace chemicals (e.g., [3]), or artificially injected heat or dyes (e.g., [4]). Alternately, flow can be approximated with a number of different mathematical methods including water budgets, hydrograph separation, Darcy's law, and numerical modeling (e.g., [5]). Regardless of the approach taken, the time-varying and spatially variable nature of the water movement favor the use of methods that are cost-effective enough to allow deployment of multiple sensors, and that provide continuous automated measurement. Manual measurements cannot provide the necessary resolution. Existing automatic sensors are expensive to build and maintain, and require frequent servicing, preventing effective deployments in winter climates when ice may form on the water surface.

One approach to the measurement of SGD and SGR is to continuously measure the vertical hydraulic head gradient within the sediment underlying a water body of interest. Darcy's law allows the vertical water flux density, q_z_ (LT^-1^), between a sediment-water interface and a point at depth *L* below the interface to be determined when the differential pressure (DP) and the hydraulic conductivity between the two points is known.

In the vertical direction, Darcy's law is expressed as:

(1) $$q_{z}=-\frac{k_{z}}{\mu }\left(\frac{\partial P}{\partial z}+\rho g\right)$$

where *k_z_* = permeability in the vertical direction (L^2^), *P* = pressure (ML^-1^T^-2^), *ρ* = density (ML^-3^), *g* = gravitational constant (LT^-2^), *ρ* = viscosity (ML^-1^T^-1^), and *z* = elevation (L). Integrated from depth *L* to the sediment surface and assuming only vertical flow, Darcy's law can be expressed as:

(2) $$q_{z}=-\frac{k_{z}}{\mu }\left(\frac{P_{0}-P_{L}}{L}+\frac{g}{L}\int_{-L}^{0}\rho_{s}dz\right)$$

where *P_o_* = fluid pressure at the seafloor (depth of zero), *P_L_* = pressure of groundwater at a depth of *L* below the seafloor, *ρ_s_* = density of pore water in the sediment, which is a function of elevation *z*, and ${\bar{\rho }}_{s}$is the mean of the density *ρ_s_* between the seafloor and depth *L*.

Thus, using Equation 2, changing flow can be calculated from time series of differential pressure, along with measurements of temperature and electrical conductivity sufficient to calculate the density and viscosity of the water in the subsurface and in the water column. Conductivity and temperature can be used to calculate salinity with the Practical Salinity Scale [6], from which density can be calculated using the International Equation of State of Seawater [7]. Viscosity can then be determined from the constitutive relationships presented by Langevin *et al.* [8].

We describe the technical aspects of a new instrument that can perform the above measurements continuously in the coastal zone, and can make the measurements accessible in real time through a wireless connection.

### 1.2. Instrument design objectives

The specific research questions that motivated the development of the instrument relate to the bidirectional exchange of water between coastal aquifers and oceans, particularly to understanding its controlling mechanisms at time scales ranging from tidal to seasonal. Our design objectives were driven by the conditions at our field site (Waquoit Bay, MA), but should allow for use in diverse applications including studies of surface-groundwater exchange in lakes and rivers.

Theoretical considerations along with an extensive dataset of manually measured seafloor fluxes at Waquoit Bay led us to expect vertical pressure gradients on the order of tens of millimeters of water column, or thousands of pascals, over a depth of one meter into the subsurface. Thus, the target for differential pressure accuracy is ±0.5 mm water, over a full scale range in total pressure of ±200 mm of water, with a sampling interval shorter than once per hour (i.e. several times per tidal cycle).

Furthermore, in coastal groundwater systems, significant spatial and temporal variations in salinity exist because of the dynamic mixing between fresh groundwater and seawater at time scales ranging from tidal to seasonal. Thus, it is critical that our instrument measures the temperature and conductivity of both groundwater and surface water regularly to allow for accurate flux calculations. Starting from the same error threshold for differential pressure stated above, the target for density uncertainty is a maximum of ±0.05%, or ±0.5 kg/m^3^, which in a marine system corresponds to a maximum salinity uncertainty of approximately 0.6 psu (practical salinity units). Determination of salinity to this degree of accuracy on the basis of temperature and conductivity measurement imposes accuracy requirements of approximately ± 1°C and ±1 mS/cm, respectively. The direct effect of temperature on density imposes less restrictive requirements on temperature accuracy (order of ±3°C).

It is also necessary for the instrument to be compatible with seawater, and be low in cost so that a fleet can be built on a reasonable budget. A low power budget is necessary to allow long deployments with small and relatively inexpensive battery packs. The ability to obtain differential pressure data in real time is also a design objective. Buried data cables are a possible means to this objective, but the activity of shellfishermen, boaters, and breaking waves (as we experience at the Waquoit site) render this approach problematic, and favor use of a radio link via buoys tethered to the DP loggers to elevate the antennas above the water surface. A range of at least 100 meters is needed, and cost is again an important factor.

## 2. Conceptual Overview of the instrument and its operation

**Figure 1.**
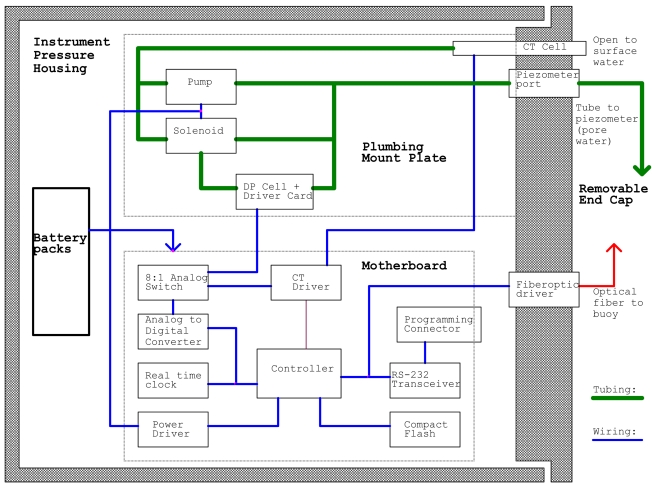
Functional block diagram of the DP logger showing major fluid flow and electrical circuits.

The concept for this instrument is illustrated by the functional diagram shown in Figure 1. Head difference is measured between two ports. The first port is open to the overlying surface water. The second port is connected directly to the sediment groundwater through a piezometer. The piezometer is a small sampling well manufactured by MHE Products [9]. It is functionally a 36 inch long metal tube that is inserted into the sediment, with openings and a filter screen at the bottom of the tube to allow the pressure at depth to be measured. A differential pressure (DP) cell is normally connected between the surface water and sediment groundwater ports. A 3-way solenoid valve can connect the two inputs of the differential pressure cell together, to check the zero-differential-pressure baseline. A pump is used to draw surface water and groundwater through a combined conductivity and temperature (CT) cell, providing data from which the density of the waters can be calculated.

The instrument is deployed in a pressure case and anchored in place on the sediment surface, as shown in Figure 2. A wireless data link can be attached to transmit data to shore in real time via buoys that contain wireless devices and provide stable buoyant platforms to support radio antennas above the water surface. There are several purposes for such a data link, including monitoring for instrument malfunctions and checking battery status as well as identifying significant episodes of, e.g., high head differential that could trigger immediate supplemental measurements, such as manual sampling for chemical concentrations.

During operation, at each sampling period the DP cell output is averaged over several samples and logged. Then the solenoid valve is operated such that the two ports of the DP cell are connected together, and the resulting reading is logged, to allow for correction of zero offset. Several times a day, the pump is run for approximately 10 seconds to pull surface water into the CT cell, so that the conductivity and temperature of the surface water can be measured. Then the pump is reversed, and run for several minutes to completely flush all the tubing from the piezometer to the CT cell with new groundwater. Conductivity and temperature of groundwater are then measured and logged. Once the pump is shut off, there is no path for the surface water and groundwater to mix within the instrument, so the groundwater will remain unchanged in the piezometer until the next time the pump is run.

## 3. Detailed Design Criteria: Accuracy, precision, and the error budget

### 3.1. Pressure Error

The maximum acceptable differential head error at our study site at Waquoit Bay on Cape Cod, Massachusetts is estimated to be ±0.5 mm H_2_O. The following equation defines the response of a real-world sensor:

(3) $$R_{A}=a\text{DP}+R_{0}$$

where *R_A_* is the actual response of the sensor to *DP*, the applied physical pressure signal, *a* is the gain of the sensor, and *R_0_* is the reading that the sensor gives with no applied signal. Ideally *a* and *R_0_* are both unchanging; however, both do vary with temperature, orientation, aging, and common mode pressure. Additionally, *a* can vary with changes in *DP*, which is referred to as non-linearity. We refer to changes in *R_0_* as zero or offset errors, and changes in *a* as gain or slope errors. Any changes in *R_0_* that are uncorrelated with any other physical parameters are considered noise if they are rapidly varying with respect to *DP* and drift if they are slowly varying.

In selecting a differential pressure transducer, it became clear that a sensor which displayed sufficiently small errors, particularly drift, would be excessively costly, physically large, and consume too much power. Worse, even the most expensive sensors had significant sensitivity to orientation. The orientation and motion of a sensor has an effect on the reading, due to the non-zero thickness of the diaphragm and the difference in density between the diaphragm and the pressure media. This error is seen as a zero offset shift, proportional to the thickness of the diaphragm, the degree of tilt (i.e. the component of the acceleration of gravity that is normal to the diaphragm), and the density of the fluid involved. The effective thickness of the diaphragm in a stable and rugged sensor is typically several centimeters. Thus, the resulting design is oriented toward mitigating the above types of error and making a small, inexpensive sensor perform adequately for our task.

### 3.2. Conductivity and temperature measurement error

Unlike the case of differential pressure measurement, sampling of the conductivity and temperature of water requires that water be pumped through the sensing cells. While, for this purpose, errors due to small uncertainties in flow rate are not significant, it is necessary that the temperature and electrical conductivity of the water be measured with sufficient precision and accuracy.

It is not difficult to meet the ±1°C criterion for temperature accuracy. However, conductivity cells under long-term deployment can be problematic due to formation of biological fouling on the electrode surfaces, corrosion of the electrodes, as well as, potentially, the development of electrical leakage paths. The former problem can be mitigated by use of a four-electrode system in which the sensing electrodes are part of a high-impedance circuit which is minimally affected by high-resistance surface coatings. However, there is a trade-off between cost and simplicity. In the original implementation a relatively low-cost commercially-available conductivity flow cell was implemented, and means were taken to mitigate errors as described below. In retrospect this cell proved to be a poor choice, and is a weakness of the instruments which we are taking steps to eliminate.

### 3.3. Fluid system error

The largest practical concern in the fluid system is the possible presence of air bubbles that occupy the full cross section of the fluid circuit. Air has a negligible density compared to water; so for example, a one millimeter tall air bubble in a vertical fluid line will result in an error of one millimeter of head. Error due to one such bubble alone would exceed the allowable error budget of the entire instrument. Moreover, offset errors created in the fluid system will manifest as an apparent differential pressure signal, so auto-referencing the DP cell will not correct for them. Thus, the utmost care was taken not to introduce bubbles into the system. We have used de-aired water when assembling the fluid system to minimize the possibility of bubble formation. Wherever possible tubing and components are filled with water before being mated. Clear tubing and fittings are used extensively to make bubbles easy to locate visually. Once the system is fully assembled, a vacuum is pulled simultaneously on both pressure ports to reveal any small air bubbles that might not go unnoticed otherwise. Air bubbles are coaxed out by a combination of running the pump to move water through the tubing, rotating the fluid system to let gravity help, and by gently tapping on the tubing to dislodge bubbles. Once deployed, a benefit of using a pump to move water periodically is that it helps to remove any small air bubbles before they can coalesce and grow.

### 3.4. Signal processing error

Errors can also occur after signal transduction has occurred, causing the signal output by the sensor to be processed or recorded inaccurately (i.e. electronic errors). In contrast to the challenges of minimizing or compensating for sensor and fluid system error, the design of electronics to accurately amplify, digitize and record signals with accuracy in the one part per thousand range is relatively straightforward. It is further facilitated by the fact that any small offset errors introduced by the circuitry are automatically corrected, along with sensor offset error, by the auto-referencing procedure. Nonetheless, the achievement of adequate accuracy and precision does call for good practices to maximize stability of the electronics and minimize effects from low battery status as well as from changes in temperature, or other environmental factors. Measures taken to assure that signal processing error is not significant in the DP logger are discussed in more detail below.

## 4. Materials and Methods

### 4.1. Key components

Several aspects of the DP logger design are tightly tied to the specific choice of certain key components. The several component choices that bear most heavily on subsequent mechanical and electrical design are discussed below.

#### 4.1.1. Pressure transducer

Perhaps the single most critical component of the DP logger is the differential pressure cell. The Honeywell 24PC pressure sensor was chosen for the instrument. Among its advantages are its low cost and small size; it is less than 1.3 cm in any dimension, excluding the ports, and weighs about 2 g. All components in contact with the water are plastics with excellent corrosion resistance. The pressure sensor can tolerate a considerable overpressure, and it has a very small power requirement. In addition, due to the small size of the diaphragm, the effect of tilting the sensor on the pressure signal is very small, as opposed to the larger and much more expensive sensors discussed earlier, for which tilting can create a considerable error. A summary of key specifications of the 24PC is shown in Table 1.

Despite the advantages, several challenges had to be overcome to effectively utilize the 24PC sensor. First and foremost, it can be seen in Table 1 that the specified zero offset error of the sensor considerably exceeds the requirements imposed on the instrument. Moreover, sensor output is subject to drift with time and temperature.

Using a solenoid valve to auto-reference the sensor eliminates, for practical purposes, the zero offset effects, from whatever cause. However, it does not address sensitivity errors such as arise from temperature change. These can amount to 5% of the span, or 0.5 mm on a 10 mm applied pressure. While resistive bridge sensors such as the 24PC are typically driven by a constant voltage, in this case the gain of the sensor exhibits a serious dependency on temperature. Instead, we follow Honeywell's recommendation and drive it with a constant current. This technique provides reasonably good temperature compensation. As the temperature increases, the pressure sensitivity (ΔR/ΔDP) of the piezoresistors decreases, but the total resistance increases. When driven by a constant current, the increasing resistance increases the voltage on the bridge, thus compensating for the reduced pressure sensitivity. An additional benefit to this method is that the voltage across the bridge is a reasonable gauge of the temperature of the sensor itself. Any remaining span shift due to temperature, as well as the non-linearity of the sensor, can be quantified and corrected in post processing by careful calibration of the DP cells at several temperatures.

The Repeatability and Hysteresis specification lies just at the limit of acceptable errors. However, neither presents a serious error in practice. Repeatability is essentially the noise of the sensor. As such it is randomly distributed around a mean value corresponding to the actual applied pressure, so averaging several readings will greatly reduce the magnitude of repeatability error. Hysteresis is the difference between the reading obtained when a particular pressure has been approached from a lower pressure, and the reading obtained when that same pressure has been approached from a higher pressure. Since the solenoid valve applies a zero pressure condition across the sensor at every sampling cycle, every pressure reading will be approached from zero. Incorporating the solenoid valve in the calibrations ensures that the effect of hysteresis is also accounted for in their results. The manufacturer's non-linearity specification is only for positive pressures, hinting at an increased non-linearity across the zero crossing. Non-linearity errors are repeatable, and can be included in calibration curves if necessary.

#### 4.1.2. Temperature/conductivity cell

In the original instrument design, the conductivity and temperature cell used was the model 545 flow-through cell made by Amber Science. It is a small tube, approximately 12.7 cm long and 1.1 cm in diameter, with two platinum plated electrodes and a thermistor at one end. We designed custom electronics to drive the cell in order to keep power consumption down and simplify the interface. Conductivity and temperature are only measured during pumping. At all other times driving electronics are shut off, and completely disconnected from the conductivity cell and thermistor by analog switches. This is intended to save power as well as improve the life of the electrodes by protecting them from galvanic corrosion.

After data from field deployments demonstrated the vulnerability of the 545 cell to corrosion, we replaced it with the model 858 cell, also by Amber Science, which uses 316 Stainless Steel plates as electrodes and was shown to be resistant to corrosion during field deployment. The same driving electronics were used for the 858 cell, although the pumping durations in both forward and reverse directions for sampling groundwater and surface water conductivity and temperature had to be increased due to its larger internal diameter (0.36″ or 0.9 cm) and length (9.65″ or 24.5 cm).

The larger size of the 858 cell represents a weakness in the current instrument design, because of the resulting larger pumping durations and hence increased power consumption. Nevertheless, the use of this cell allows us to deploy our instruments and collect accurate conductivity and temperature data, while simultaneously developing a new low-cost 4-electrode, corrosion resistant conductivity sensor.

#### 4.1.3. Fluid circuit components

A peristaltic pump from Autoclude was chosen for several reasons, including its simplicity; it comprises only a DC electric motor driving two rollers in a circular motion around a flexible tube inside a plastic housing. The only wet component is the interior of the tube. Flow is easily reversible simply by changing the polarity of voltage applied to the motor. The volume of water displaced by the pump in a given time is a known function of voltage applied to the motor. Further, when the pump is off, it presents no path for flow of fluids, allowing it to be placed across the differential pressure transducer without interfering with the pressure signal. The pump can handle some small solid particles in the tubing without trouble, in case a little sand or sediment is sucked in. Servicing the pump is easy and inexpensive. The tubing can be quickly replaced when it becomes worn.

The solenoid valve used is a poppet valve from Neptune Research. It is small and relatively inexpensive. The wet parts of the valve are PTFE (Teflon®), so it is compatible with salt water. The mission-average power requirements are low, given that it needs to be energized for only a few seconds each time a sample is taken. In practice, 12 standard alkaline D-Cell batteries provide enough energy to run the solenoid valve for a sample every 5 minutes for a year. The pump, solenoid valve, DP cell, and conductivity cell are shown in Figure 2.

#### 4.1.4. Data Link to shore

Wireless data links to a shore-based PC are based on Handy-Port HPS-110 OEM transceiver boards. These boards run under Bluetooth and are powered at 4.5 volts. They receive data via an RS-232 compatible serial port and transmit at a power level of approximately 16 dBm. The manufacturer-supplied antenna is a half-wavelength antenna providing 2 dB of gain. Data are received at a remote PC or laptop computer located on shore and equipped with a single Handy-Port HPU-120 wireless transceiver. Maximum range over water is in excess of 300 m, and the wireless link is bidirectional, although we have to date only provided a unidirectional fiber optic connection to transmit data from DP logger to buoy. In the current configuration the rf link is maintained constantly.

Because of the cost of underwater cabling and penetrators, as well as concern about the fatigue of metallic conductors that could be employed to connect the radio-equipped buoys to the DP loggers, we chose a low-cost jacketed plastic optical fiber (model SH4001 from Industrial Fiber Optics) to transmit data from the DP loggers to the buoys.

### 4.2. Mechanical Design

#### 4.2.1. Differential Pressure Logger Housing

The mechanical design of the instrument is optimized for deployments in shallow, energetic waters. It is small, relatively light, and easy to install and remove. The weight of the instrument fully loaded with batteries is approximately 19 kg. However, the underwater weight of the instrument is only about 5 kg due to the volume it displaces. After it is installed in the deployment location, lead weights can be tie-wrapped onto it to reduce wave induced motion.

The electronics, sensors, plumbing, and batteries are enclosed in a Poly-vinyl Chloride (PVC) water tight pressure case which is machined from a length of standard 6 inches (15.25 cm) schedule 80 PVC pipe. One end of the cylinder is sealed with a solid 1.9 cm disc of PVC plate glued in place and further sealed with polyurethane sealant on the outer seam. The opposite end is machined to accommodate a removable end cap with o-ring seals which is held in place with machine screws. The assembled housing is rated for up to 300 psi of water pressure, or approximately 200 meters deployment depth.

**Figure 3.**
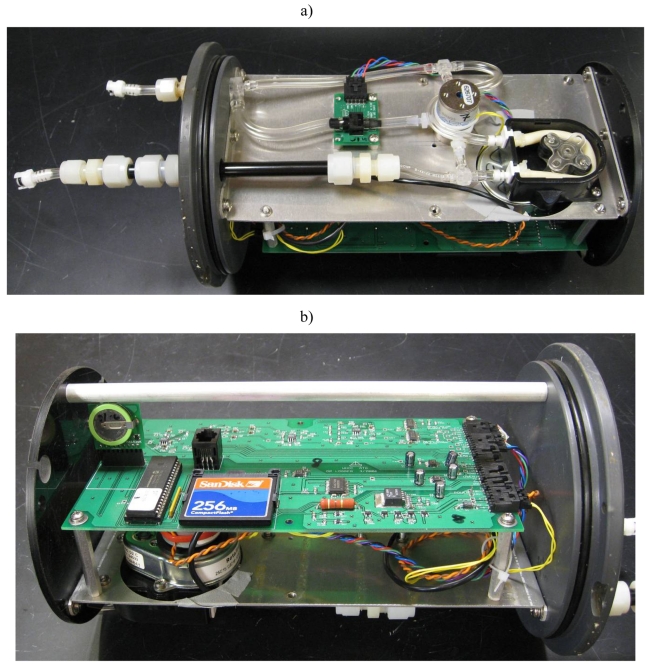
a) The internal fluid system mounted to the chassis – clockwise from upper left: groundwater port, DP cell with driving circuit board, solenoid valve, peristaltic pump, 545 CT cell feeding through the end cap to the surface water port; b) The motherboard of the logger mounted to the instrumentation chassis. The small vertical circuit board on the far left is the real-time clock. Next to the clock is the PIC processor, then the Compact Flash card. Beneath the processor, the motor for the peristaltic pump can be seen. The connectors on the right side of the board handle all interconnections to batteries, sensors, fluid components, and the user serial port.

The removable end cap is machined from a 1.9 cm thick plate of PVC. There are two ports drilled through to accommodate the flow of water in or out of the instrument, and a third port to accommodate the optical fiber for the communication buoy. Attached to the low pressure side of the end cap is an instrumentation chassis and battery compartment, which provides a means of mounting the electronic circuitry and fluid system components and which is easily removed from the housing for bench testing and calibration. Figures 3a and 3b show the two sides of the instrumentation chassis.

**Figure 4.**
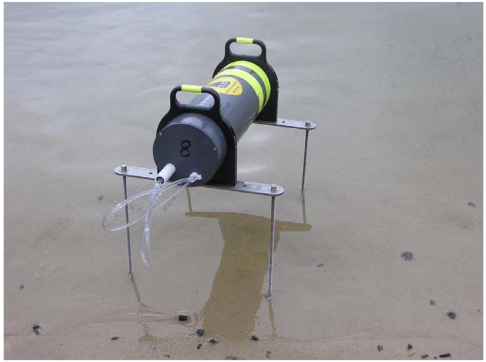
An early differential pressure logger ready for deployment. This instrument has not yet been upgraded to include a port for wireless communication.

The assembled pressure housing is mounted to 43 cm × 5 cm × 0.6 cm thick type 316 stainless steel mount plates with two clamps machined from 1.9 cm black acetal plate. The clamps are made up with an integral plastic handle on the top side to make it easy to carry. On the outer ends of the stainless plates there are 0.95 cm type 316 stainless threaded rods bolted through, which are intended to be pressed into the bottom substrate to anchor the instrument in place during deployment. An assembled differential pressure logger is shown in Figure 4.

#### 4.2.2. Fluid circuit

Standard 0.32 cm internal diameter (ID) clear PVC flexible tubing, with polycarbonate barbed fittings for couplings, is used for most of the plumbing of the DP logger. The two pressure ports are sealed by nylon Swagelok fittings screwed into NPT threads in the PVC end cap. The ports are terminated by luer-lock fittings to allow connections to be easily made and broken in the lab, field, and underwater. The piezometers we have used in Waquoit Bay are model PPX36 by MHE Products. These are 0.91 m in length with a 1.1 cm ID. A stiffening rod is inserted into the piezometer when inserting it into the sediment to keep it from bending. Once the piezometer is installed, this stiffener is removed, and replaced by a semi-rigid 0.32 cm ID tube, secured and sealed to the top of the piezometer by a Swagelok fitting. This tube reduces the ID of the piezometer to shorten pumping times and save power. A luer-lock fitting inserted through the Swagelok allows for connection to the appropriate pressure port on the DP housing end cap. When the instrument is not deployed a tube is used to connect the two pressure ports on the end cap together. Ideally this tube is only removed when the instrument is immersed in water, and then reinstalled before removing the instrument from water. This keeps air bubbles from forming in the plumbing, as well as providing a low pressure path for water to flow through if the pump has a scheduled activation while the instrument is not submerged in water.

#### 4.2.3. Buoy mechanical design

**Figure 5.**
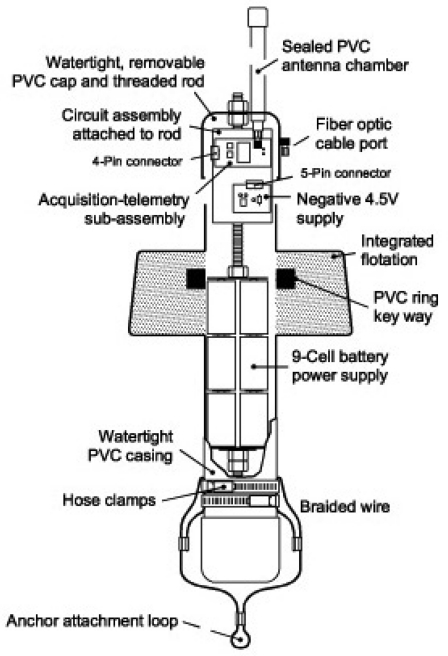
Cutaway drawing of a data buoy showing arrangement of major components. Overall height of buoy is 50 cm, exclusive of antenna. Note that top cap with attached electronics boards and battery pack is illustrated as being lifted partway out of the body of the buoy.

Buoy housings are constructed of nominal 3 inch (7.6 cm) schedule 40 PVC pipe, to which a standard cap is glued at the bottom end. A rack to hold electronic components, including the battery, is mounted to a second standard PVC pipe cap that is mounted on the top end of the buoy. The battery is mounted at the bottom of the rack, to provide for the lowest possible center of gravity of the assembled buoy. The wireless board is mounted at the uppermost end of the buoy so that the half-wave antenna will have maximum vertical height when connected directly to the RF output connector on the board. To provide necessary additional buoyancy and stability, a toroidal molding of buoyant foam (US Composites ‘8 pound’ foam) is cast to the top end of the buoy, facilitated by a mold built from an angel food cake pan and coated with mold release (Partall paste wax, part number REX224). A diagram of buoy mechanical construction is shown in Figure 5

### 4.3. DP Logger Electrical Design

#### 4.3.1. Analog Electronics

Several measures are taken in the ‘front end’ to minimize both noise and systematic error associated with the differential pressure cells per se. Each pressure sensor is soldered to a small circuit board containing all the electronics necessary to drive it and amplify the output. This has several advantages. First, noise is reduced by amplifying the millivolt output of the sensor close to the source. Second, the electronics can be tailored to the individual sensors; by selecting appropriate values for one resistor on the board the span of the sensors can all be made to be within 1 to 2 percent of one another. Otherwise, the gain from one sensor to the next can vary by as much as a factor of two. Third, each driving and amplifying circuit is uniquely tied to one sensor, so when the sensor is calibrated, the calibration will take into account variance among the electronic circuits.

To drive the conductivity cell, a resistive divider generates a 50 mV signal that is buffered by operational-amplifiers. Analog switches transform this signal into a square wave. The square wave is fed onto the conductivity electrodes and the resulting current is proportional to the conductivity of the seawater in the tube. Driving the cell with an AC signal eliminates errors caused by build up of ions around the electrodes associated with DC measurements, as well as reducing electrolytic corrosion. The analog switch is configured so that it can accept either a two or four electrode conductivity cell. Using a four electrode cell would bring some benefits; in the four electrode configuration, two electrodes are used to drive the current through the water, but the voltage is sensed between the other two electrodes. Essentially no current flows through these sense electrodes, eliminating the effect of resistance imbalances from the analog switches, circuit wiring, fouling of the electrodes, etc. The thermistor is used as one half of a voltage divider to create a temperature signal. Careful selection of the other resistor ensures a nearly linear relationship between temperature and output voltage.

Signals from the differential pressure cells and conductivity-temperature cells are digitized using a Linear Technologies LTC2400 analog to digital converter. This is a micropower 24-bit Delta-Sigma converter that consumes only 200 μA during conversion, and much less than that in sleep mode. Communications are handled over the same I2C bus used for the real-time clock. An 8-input multiplexer is used to switch the various signals to the A-to-D one at a time. In addition to the differential pressure, temperature, and conductivity measurements, the die temperature of the differential pressure sensor is measured, the three battery voltages are measured, and a leak detect circuit provides early warning of water intrusion in the pressure housing via the wireless ink. The leak detector comprises two wires held at different electrical potentials and physically located close to but not touching the bottom of the housing. If water bridges these two wires, current will flow, generating a signal across a 1 megohm series resistor. The sensitivity is sufficient that even fresh water will create a noticeable signal.

Throughout the system certain measures are taken to ensure that the design goals are met. The reference voltage is generated by a resistive divider from the regulated analog rail, and is buffered by an op-amp. All measurements are made ratiometrically so the actual value of this reference is not critical. Low power precision op-amps are used throughout to keep power consumption low. The average power consumption of the analog electronics is less than 15 mW, and is dominated by the differential pressure sensor itself. While this sensor could be switched off when not in use to further reduce power consumption, this is unnecessary, so we leave it on continuously to eliminate any possible errors caused by thermal effects of power switching.

#### 4.3.2. Digital Electronics

Control and data logging are accomplished digitally using a PIC18LF877 processor, manufactured by Microchip Technology Inc, chosen for its versatility, ease of programming, low power and low cost. It is run with a 614 kHz clock, generated by a Linear Technologies LTC6906 programmable silicon oscillator. The power consumption of the processor and oscillator at this clock speed is less than 200 μA. The processor's on-board universal asynchronous transmitter/receiver (UART) is used in conjunction with a Maxim MAX3223 RS-232 transceiver to enable communication between the instrument and the operator. An adaptor cable connects the board to a standard serial port, through which the operating parameters can be programmed, the clock can be set, and data can be observed in real time for testing or calibration. The RS-232 driver increases the power consumption of the board by an order of magnitude, so it is kept switched off except when an active RS-232 link is detected. The 3V logic-level signal from the UART is also taken off board to the fiber-optic link to the wireless buoy.

A Maxim MAX3231 real-time clock (RTC) is used to provide timing for the acquisition system. This is a single-chip highly integrated package, containing a silicon oscillator, a temperature sensor, an array of switchable capacitors for temperature compensation, and the logic circuitry of the RTC. Over the temperature range in the field (0–40°C) the clock keeps time to within ± 2ppm, or about 1 minute per year. The devices can be recalibrated as necessary with a frequency counter. We package the surface mount part on interchangeable postage-stamp-sized boards to allow the clocks to be calibrated independently and swapped out if necessary. The board also holds a lithium coin-cell battery to backup the time and settings on the clock. Communication with the PIC is handled via the I2C protocol. The real-time clock generates different alarms to control the sampling. It can be programmed with a start time and date to allow the operator time to seal up the instrument, transport it to the field and install it. When the alarm goes off, the PIC switches to normal run mode and takes its first reading. In normal run mode, the RTC generates an alarm every minute, on the minute. The PIC checks the time, and if it is the appropriate time it will take a sample, generating a timestamp from the RTC time. The RTC can also be configured to generate an alarm once every second, for more frequent sampling during laboratory calibrations.

A standard Compact Flash card is used for data storage. These cards have many advantages – the cards and readers are readily available, very inexpensive, and even the smallest cards available today have enough capacity for years of data in this application. The PIC formats the cards with a basic FAT16 file system, which all major operating systems can recognize. It creates a boot directory, a file allocation table, and a root directory containing two file entries. One file allows the user to enter a short text information file containing information about the deployment. Data is collected into the second file as plain ASCII, in a space separated values list, one sample per line. Downloading and analysis of the data is thus very straightforward; a text editor can be used to quickly inspect the data, and the data can be imported directly into applications such as Excel or Matlab for further processing.

Using the serial interface, all of the components of the instrument can be controlled manually to facilitate testing and calibration. Additionally, various parameters can be set to change the behavior during deployments. These parameters include the interval between samples (1-60 minutes), the interval between running the pump to check conductivity and salinity (1-24 hours, or not at all), and the duration of pumping (1 minute up to the sample interval). The parameters will allow the instrument to function in a wide range of environments with minimal modifications. Two more run modes are available for short term testing where power consumption is not a concern: “fast mode”, and “calibration mode”. Fast mode is similar to the standard run mode, in that the solenoid valve is actuated at intervals, and the pump is run at longer intervals, but the instruments are recording loosely timed data continuously. This provides information on the dynamics of a location, for instance how much effect wave motion has on the pressure sensor and how long the pump must be run to draw groundwater into the conductivity cell. Calibration mode, on the other hand, is designed for use in the lab to calibrate sensors. It takes temperature, conductivity, and pressure readings at precisely timed intervals (1-60 seconds), but does not run the pump or solenoid.

#### 4.3.3 Power

The motherboard runs from a battery pack consisting of 12 D-cell batteries which can be either alkaline, for economy and safety, or lithium, for maximum longevity. A separate battery pack consisting of 48 D-cell batteries is used to run the pump and solenoid valve. Conservatively, an alkaline electronics pack will last approximately six months, giving a long service interval. The operational lifetime of the pump pack depends on how frequently the pump is used, but in general will also last around 6 months with ordinary usage. If one pack is exhausted well before the other, it can be replaced independently.

### 4.4. Data Link and Buoy

**Figure 6.**
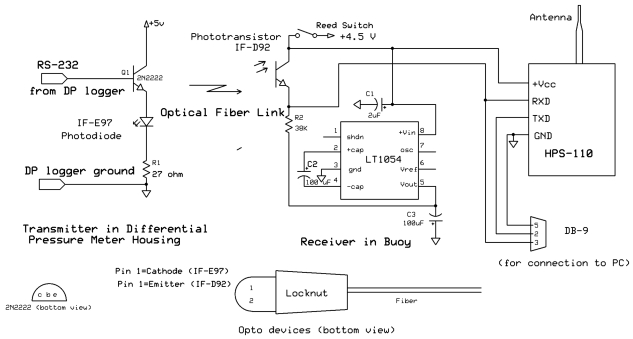
Circuit diagram for the wireless data buoys, including fiber optic link to DP logger and optical transmitter incorporated into the DP logger. The physical packages of the optical transmitter (LED) and receiver (phototransistor) are identical, and each includes a locknut for holding the plastic-jacketed optical fiber (see above).

A circuit diagram for the buoy and fiber optic data link is shown in Figure 6. The unidirectional link from a DP logger to a buoy is provided via 1 mm diameter plastic fiber having a black outer jacket (Industrial Fiber Optics SH4001). This fiber has an index of refraction of 1.49, a numerical aperture of 0.5, and is rated for an endurance of 10,000 bends of 25 mm radius. A red (670 nm) transmitting LED unit (International Fiber Optics IFE97, which comprises both an LED and a compression nut for receiving and holding the end of an optical fiber) is driven via an emitter follower by the serial signal from the UART of the microprocessor. The transmitting LED unit is encapsulated into a length of nominal ¼ inch PCV pipe nipple using West System epoxy resin, and the resulting feed through assembly is threaded into the end bulkhead of the DP logger pressure case. The optical fiber for connection to the buoy can be field-cut with a razor blade, and optical fiber connections to the DP logger can be assembled underwater using the plastic compression nut of the IFE97 LED unit.

At the buoy end of the optical fiber the signal is received by an IF-D91 phototransistor. When light is received, the transistor conducts, pulling the RXD input of the HPS-110 wireless board's serial port to the ‘high’ logical state. Between light pulses the transistor does not conduct, and the RXD input is pulled negative by the -4.5 volt supply through R2. This negative voltage level is required to express a ‘low’ logical state to the serial input. The buoy operates from a +4.5 volt supply provided by the buoy's battery, with the negative supply being provided by an LT1054 integrated circuit from Linear Technologies. The LT1054 uses a flying capacitor circuit to produce an output of essentially the same magnitude, but opposite polarity, relative to its +4.5 volt power input.

The reconstructed signal that is applied to the RXD input of the HPS-110 wireless board falls within the receive specifications of the RS-232 standard. A DB-9 connector is also provided within the buoy housing so that communication parameters for the board can be set via a serial connection using a terminal program on a computer. The optical link and its associated RS-232 input to the board is currently operated at 1,200 baud, while the wireless network itself is typically set to run at 4,800 baud. These rates greatly exceed the rate of data collection by the DP loggers.

Power to the HPS-110 board as well as to the -4.5 volt supply board is provided at 4.5 volts from a series-parallel arrangement of nine alkaline D cells. Power can be turned on or off without opening the buoy housings by means of a magnetic reed switch and a magnet that is applied to the outside of the housing in the vicinity of the switch.

### 4.5. Laboratory Calibration

#### 4.5.1 Differential Pressure Sensors

Prior to instrument construction and before field deployment, the differential pressure cells are calibrated in the laboratory. In the calibration experiments described here, a glass column of length 20 cm and internal diameter 2.5 cm is connected to each port of the differential pressure sensors using PVC tubing filled with de-aired distilled water. All differential pressure sensors are calibrated simultaneously by connecting them in parallel to the two water columns, so that they are all exposed to exactly the same pressure variations and calibration conditions. A three-way solenoid valve is included in the calibration setup to ensure cancellation of any drift in the null output of the sensors over the duration of the calibration exercise. Precisely known forcings of differential pressure are created by starting from zero DP and pipetting a corresponding volume of distilled water into one of the columns (given the dimensions of the columns used, addition of 0.5 mL of distilled water produces a pressure increase at the sensor port of 1.02 mm of H_2_O). The initial zero DP is achieved by temporarily opening a flow path between the two columns until the water levels in them equilibrate. Following this method, the response of the DP loggers to various applied differential pressures over the range of interest is measured. Applied forcings are also repeated within a calibration exercise to quantify the repeatability errors of the sensors. A linear calibration curve is fitted to each sensor's response to positive and negative applied differential pressures separately, to avoid an evident non-linearity in the sensor response near zero DP and to highlight any non-symmetry in the calibration results.

#### 4.5.2. Conductivity-Temperature Cells

All CT cells are calibrated simultaneously by placing them together in a bath of saline water along with a reference CT cell (WTW TetraCon 325), and varying the temperature of the water bath over the range 2 °C to 27 °C using a temperature-controlled chamber. To ensure that conditions are uniform within the water bath, we vary the temperature in the chamber in steps of 5 °C and maintain a constant temperature for three hours at each step, to allow the water temperature of the bath to equilibrate with the chamber temperature. Furthermore, we cover our temperature range in both increasing and decreasing directions to ensure that hysteresis errors are quantified in the calibration exercise. The conductivity and temperature signals of the CT cells during periods of stable water temperature are compared to those of the reference cell to obtain calibration curves for each cell's temperature and conductivity response. The temperature variation covered in our calibrations is representative of conditions at our field site, Waquoit Bay. Similarly, the range of conductivities observed at our field site was covered in calibrations by using two water baths, the first with a conductivity of 27 mS/cm at 25°C and the second with a conductivity of 46 mS/cm at 25°C. Additionally, the accuracy of the reference CT cell was regularly tested using a standard solution of 30.1 mS/cm at 25°C, a conductivity which falls at the midpoint of our range of interest.

## **5.** Results

### 5.1. Differential Pressure Cell Calibration

**Figure 7.**
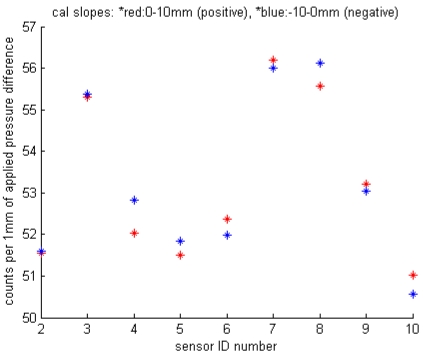
Sensitivity (slope) of each of 9 DP loggers. These data include any effects of variance in signal conditioning and digitization.

Several sets of calibrations were carried out at room temperature in the laboratory using the procedure previously described, with 9 differential pressure loggers at a time. The sensitivity of each of the loggers, expressed in terms of counts (A/D converter output) per mm H_2_O of pressure difference, is shown in Figure 7. Sensitivity is of the order of 50 counts per mm for all loggers, but varies enough that individual calibration of each sensor is necessary to meet the design goals. The symmetry of the calibration curves for positive vs. negative differential pressures ranged from near zero to a high of approximately 0.8 counts per mm for DP sensor #4, implying a maximum slope error of the order of 1.6% if calibrations for positive pressure were applied to all measurements. This would correspond to a maximum error of 0.16 mm H_2_O in the case of a total differential pressure of 1 cm H_2_O.

**Figure 8.**
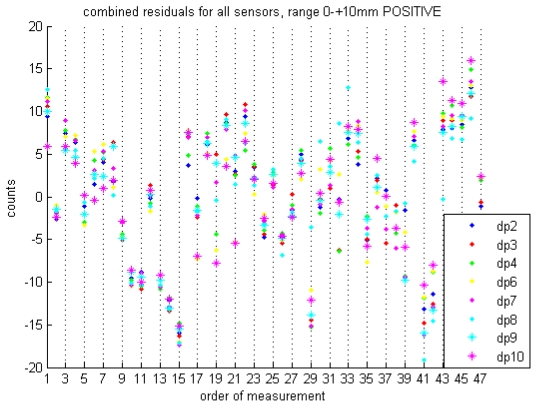
Residuals between individual measurements and a linear calibration curve fitted to all calibration data obtained during five experiments over a period of three weeks in August 2008.

Figure 8 shows the differences (residuals) between individual measurements and a linear calibration curve fitted to all calibration data obtained during five calibration experiments over a period of three weeks in August 2008. Data from each of the 9 DP loggers are analyzed independently. The vast majority of residuals are within 15 counts, or ±0.3 mm H_2_O, of zero. Data for negative differential pressure calibrations are similar. The maximum residual observed for the entire data set was 24 counts, corresponding to slightly less than 0.5 mm H_2_O.

Notice that for each measurement, the residuals of all nine sensors relative to their respective calibration curves are very close to one another (usually within five counts), even when they are outliers. This implies that either there is some systemic nonlinearity to all of the sensors, or that errors external to the DP loggers and inherent in the calibration setup account for the outliers. It seems unlikely that the first case would be true. While the sensors do display non-linearity we would not expect this non-linearity to be consistent from one unit to the next. The latter explanation implies that with a refined calibration technique, particularly one less dependent on the human element, these outliers could be eliminated and the error band around the calibration curve could be reduced.

Further calibrations are underway to quantify the variability of gain with changing temperature. Preliminary results show that the sensitivity decreases as temperature decreases, and that this effect is modest, ranging from about 1% to less than 4% for a change from 25°C to 5°C, corresponding to the typical temperature range at Waquoit Bay.

#### 5.1.1. Conductivity and Temperature Cell Calibration

**Figure 9.**
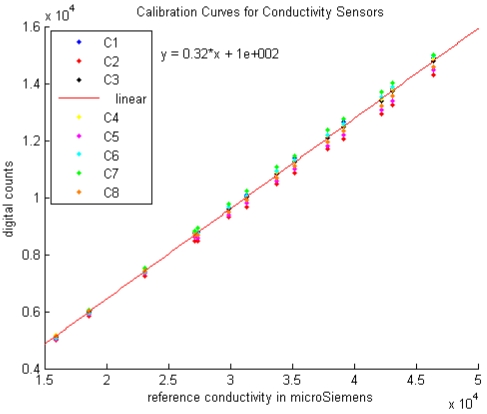
Conductivity calibrations for eight different DP loggers, with the best fit line for CT cell #3 shown in red along with its equation.

The most recent calibration data for electrical conductivity for eight different loggers are shown in Figure 9 for the range 15 mS/cm to 50 mS/cm, which encompasses the values that have been observed in the field. This data was collected using the Stainless Steel 858 CT cells rather than the original Pt-coated 545 cells, however the results for both types of cells are very similar. Calibration data were well fit with a linear equation, with slopes of around 300 counts per mS/cm. Residuals (not shown here) are nearly all less than 60 counts, corresponding to less than 0.2 mS/cm.

The temperature calibration data for the CT cells (both the 858 and 545 models) showed that the cells exhibit an adequately linear response over the range of 2°C to 27°C, with residuals remaining less than 0.3°C.

### 5.2 Field deployments

**Figure 10.**
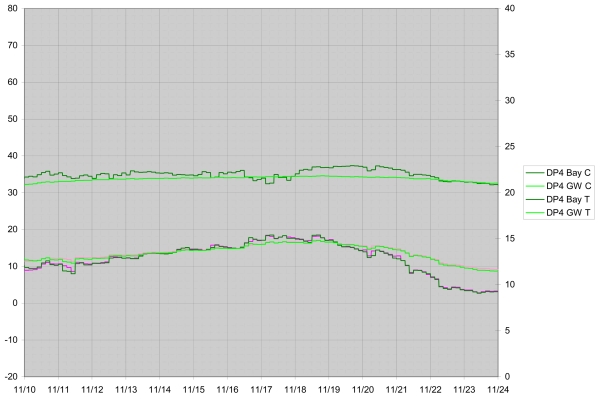
Conductivity and temperature data from one of the DP loggers deployed in Waquoit Bay, MA, in November 2006.

**Figure 11.**
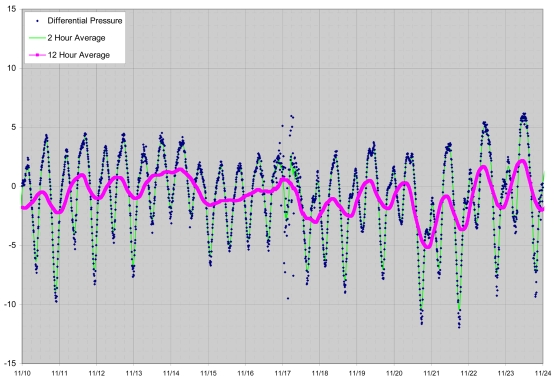
Differential pressure data, with 2 and 12 hour averages, from one of the DP loggers deployed in Waquoit Bay, MA, in November 2006.

To date eight instruments have been constructed and deployed in Waquoit Bay, an estuary on the south shore of Cape Cod, Massachusetts. Deployments occurred nearly continuously from November 2006 to June 2008 and have been largely successful. These field experiments have included wintertime deployments, deployments of individual DP loggers for as long as nine months, and deployment during which data were successfully received and logged via the wireless system at a receiving location approximately 100 m distant and inside a wood frame building. See Figures 10 and 11 for examples of data collected. Detailed results and interpretation relating to field conditions of SGD and SGR in Waquoit Bay will be presented elsewhere. Two problems requiring further instrument development have manifested during these deployments which we will discuss here.

#### 5.2.1. DP cell failures

On the first relatively long term deployment of four instruments from Nov. 9, 2006 through Jan. 25, 2007, two DP cell failures occurred. A third DP cell failure was experienced in the lab later in 2007 during calibrations. In all three cases, the silicon diaphragm ruptured and separated from the body of the DP cell, as evidenced by fragments of the diaphragm that were visible in the tubing near the cell. Without the diaphragm in place, water can flow through sensor, shorting out the driving circuitry and analog power supply.

No conclusive cause for these sensor failures has been found. The sensors are rated for an overpressure of up to 20 psi, a condition which may change the zero offset but should not cause total failure. Field conditions do not create such a magnitude of differential pressure. The pump seems unlikely to be capable of creating such pressure differential, and even if it produced a perfect vacuum, the differential with surface water at the depths of deployment would be less than 20 psi. The inertia of the pump rollers prevents it from starting or stopping abruptly, so water hammer should not be a problem. Further, the solenoid valve is engaged whenever the pump runs, preventing the pump from creating a differential pressure across the DP cell. It is conceivable that the solenoid valve could create a pressure spike when switching. However the poppet is designed to switch the connection between its ports in a make-before-break fashion. This means that there is always a path for water to flow as the poppet moves, preventing build up of pressure across the DP cell.

We theorize that the failures were in fact caused by weakening of the diaphragms resulting from rough handling of the sensors during calibration and testing, perhaps when aggressively removing air bubbles. Since adopting more cautious methods, we have not experienced any further DP cell failures in the lab or in the field, providing evidence to support this theory.

#### 5.2.2. CT cell failure

Deployments with the 545 CT cells showed their vulnerability to corrosion and the resulting effect on their conductivity signal. The failure of these cells is evidenced by the measured conductivity beginning to rise almost immediately after the instrument is deployed, eventually going off scale after several weeks. This degradation of the signal has been most evident in loggers deployed in an average water depth greater than approximately 1.5 meters. However, examination of all 545 CT cells after deployment shows severe corrosion, not only to the platinum plating but to the underlying base metal.

Despite the obvious physical damage, it is not clear why the conductivity reading from some units increases, nor why it is worse in the deeper locations. As the electrodes corrode, one would expect their electrical resistance to increase, which would cause a decrease in the measured conductivity. Water could be infiltrating the epoxy, creating electrical leakage where the two electrode wires are in close proximity. This possibility, starting at a certain pressure, could explain why the failures have only occurred in deeper locations. Alternately, perhaps, ions from a corrosion process are increasing the conductivity of the seawater.

Whatever the cause, the 545 cells do not appear to be adequate for long term use in a marine environment with the DP loggers. We can not, at this time, rule out the possibility that they are adequate in some freshwater settings, nor that our own design of the driving electronics is in some way responsible. Our driving electronics were developed specifically for this logger to provide a simple and low power interface to a wide range of conductivity and temperature cells. The design works well in the laboratory, however the isolation provided by the analog switches alone may not be sufficient to protect the thin surface plating of certain CT cells, such as the 545, from galvanic corrosion during long term deployments in seawater. Field tests of the stainless steel 858 cells show that they give stable readings in the field and are resistant to corrosion.

## 6. Conclusions

We have presented a system to measure, record and wirelessly telemeter small differential pressures between two bodies of water, as well as the temperature and salinity of each body of water. Where one body of water is surface water and the other is sediment groundwater, the rate of flow can be estimated from these parameters, even in settings where density differences arising from salinity and temperature gradients are important. This instrument has broad applications in hydrology, and could find uses in bays, estuaries, rivers, and lakes. The relatively low cost allows many instruments to be deployed across an area of interest to produce superior spatial and temporal resolution compared to alternative flow measurement techniques. Laboratory calibrations have shown that uncertainty of the pressure measurement of the order of ±0.3 mm H_2_O can be achieved with careful calibration. In the laboratory, the uncertainties of conductivity and temperature measurement are ±0.2 mS/cm and ±0.3°C, respectively.

The design goals have been met in all respects, save for the corrosion concerns relating to the CT cell. We are currently modifying the instruments to use a cell with stainless steel electrodes. Unfortunately this option is physically larger, which necessitates mechanical changes inside the pressure housing, and requires longer pumping times to flush it, in turn increasing drain on the batteries. We are simultaneously developing a novel CT sensor tailored to this application, as there appear to be no commercial products that combine small size and low cost with a 4-electrode corrosion resistant cell.

## Figures and Tables

**Figure 2.**
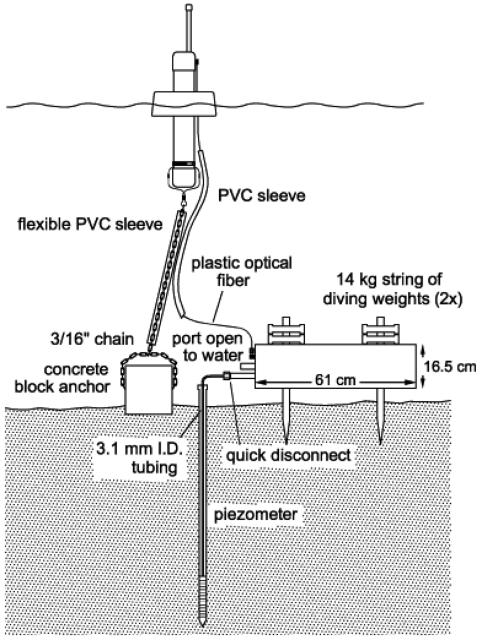
Diagram of a typical deployment of a DP Logger with wireless communication buoy.

**Figure 2.**
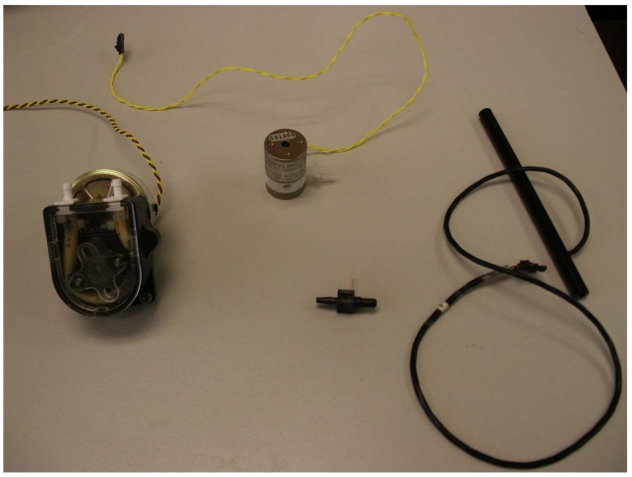
The major components of the fluid system – clockwise from left: peristaltic pump, solenoid valve, 545 CT cell (with custom connector installed), DP cell (before installation of custom circuit board).

**Table 1.** Specifications of 24PC series (Differential, Unamplified, Non-compensated pressure sensors) published by Honeywell. Performance characteristics at 10.0 ± 0.01 VDC Excitation 25°C. All values are typical unless otherwise stated.

**Pressure Range**	0.5 psi=350 mm H_2_O
**Span**	35 mV
**Sensitivity (gain)**	10 mV/mm H_2_O
**Null Shift, ΔT=25 °C**	±2.0 mV = ±20 mm H_2_O
**Span shift, ΔT=25 °C**	±5% span
**Non-linearity (P1>P2)**	±0.25% span = 0.88 mm (max ± 1.0% span)
**Repeatability and Hysteresis**	±0.15% span = 0.53 mm
**Stability over 1 year**	±0.5% span = 1.75 mm
**Response time**	Max 1 msec
**Max Overpressure**	20 psi = 14 m H_2_O
